# Active Self-Testing Noise Measurement Sensors for Large-Scale Environmental Sensor Networks

**DOI:** 10.3390/s131217241

**Published:** 2013-12-13

**Authors:** Federico Domínguez, Nguyen The Cuong, Felipe Reinoso, Abdellah Touhafi, Kris Steenhaut

**Affiliations:** 1 Department of Electronics and Informatics (ETRO), Vrije Universiteit Brussel, Pleinlaan 2, Elsene 1050, Belgium; E-Mails: cuong.nguyen.the@vub.ac.be (N.T.C.); freinoso@vub.ac.be (F.R.); abdellah.touhafi@vub.ac.be (A.T.); ksteenha@etro.vub.ac.be (K.S.); 2 Department of Industrial Sciences and Technology (INDI), Vrije Universiteit Brussel, Pleinlaan 2, Elsene 1050, Belgium

**Keywords:** sensor testing, quality assessment, environmental monitoring

## Abstract

Large-scale noise pollution sensor networks consist of hundreds of spatially distributed microphones that measure environmental noise. These networks provide historical and real-time environmental data to citizens and decision makers and are therefore a key technology to steer environmental policy. However, the high cost of certified environmental microphone sensors render large-scale environmental networks prohibitively expensive. Several environmental network projects have started using off-the-shelf low-cost microphone sensors to reduce their costs, but these sensors have higher failure rates and produce lower quality data. To offset this disadvantage, we developed a low-cost noise sensor that actively checks its condition and indirectly the integrity of the data it produces. The main design concept is to embed a 13 mm speaker in the noise sensor casing and, by regularly scheduling a frequency sweep, estimate the evolution of the microphone's frequency response over time. This paper presents our noise sensor's hardware and software design together with the results of a test deployment in a large-scale environmental network in Belgium. Our middle-range-value sensor (around €50) effectively detected all experienced malfunctions, in laboratory tests and outdoor deployments, with a few false positives. Future improvements could further lower the cost of our sensor below €10.

## 1. Introduction

Urban noise pollution sensor networks consist of spatially distributed microphones that measure environmental noise. Typical noise measurements sensors (microphones + outdoor casings) cost from hundreds to thousands of euros, rendering large-scale network deployments financially prohibitive. To counteract this limitation, high-cost noise sensors have been replaced in some network deployments with low-cost, off-the-shelf microphones. These low-cost sensors have higher failure rates and produce lower quality data, nevertheless these deficiencies can be compensated with a high density deployment, sensor redundancy and data post-processing techniques [1]. However, a high density deployment where typically hundreds of low-cost sensor nodes cover a city-wide area imply an almost unavoidable large number of sensor failures as the network ages, compromising the validity and accuracy of the collected environmental data. In this article, we define a sensor failure as a malfunction in any of the sensor's components that degrades the quality of the sensor's output data.

We developed a low-cost noise measurement sensor capable of actively testing its condition and then via the communication mechanisms available in an environmental sensor network automatically raise an alert indicating its failure. The active self-test can be triggered on demand or periodically and its result can be used to validate or invalidate the sensor's collected data. Additionally, self-tests can be used as a network maintenance tool that helps in the identification of defective sensors needing replacement. This in turn will increase the overall quality and accuracy of the noise measurement network by removing malfunctioning nodes and the contaminated data they generate.

### 1.1. Active vs. Passive Sensor Testing

Sensor testing or validation techniques can be divided in two categories: active and passive. Passive techniques rely on the analysis of the data collected by the sensor and in some cases also its neighbor sensors. They can be performed offline as a post-processing task or online in near real time. They have the advantage of not requiring any extra hardware on the sensor and are unobtrusive to the sensor data gathering task. However, they require a high degree of contextual awareness to differentiate between a sensor failure and unusual but real environmental events [2]. Common passive techniques use machine learning (Bayesian learning, neural networks, *etc.)* to detect and classify anomalies in the sensor spatiotemporal data [2,3].

Active sensor testing techniques use a stimulus that provokes a response in the sensor. This response is measured and used to estimate the condition of the sensor. They usually require extra hardware and are unique for every type of sensor. For example, a patented technique for testing magneto resistive and Hall sensors uses a coil to generate a magnetic field that excites the sensor to test its condition [4]. Another patented technique for mechanical deflection sensors uses a movable part to slightly deflect the sensor and measure its response [5].

### 1.2. Active Self-Testing Noise Sensors

Noise measurement sensors with active self-testing capabilities are only found in the high-end product lines of acoustic hardware manufactures. Brüel and Kjaer uses *Charge Injection Calibration (CIC)* to assess the quality of an environmental microphone while deployed in the field. CIC introduces a known capacitance into the amplifier circuit of the microphone and then measures its output. For a given calibration signal input, the output will change considerably in the presence of degradation or failure [6]. G.R.A.S uses *SysCheck*, which sends an extra signal (with a known voltage level and frequency) to the microphone pre-amplifier. The electronic response (capacitance, resistance) of the microphone is then measured [7]. Norsonic's environmental microphones use an electrostatic actuator placed on top of the microphone capsule. The actuator, when activated, generates an electrostatic force field that simulates an acoustic signal. The response of the microphone to this force field is measured to check the microphone's condition [8].

Currently, most active testing techniques in acoustic noise sensors are patented and products implementing them are usually prohibitively expensive for large-scale high-spatial density deployments. Our active self-testing sensors were deployed in the context of the IDEA project, an environmental measurement network operating in Flanders and Brussels (Belgium) since 2010 [9]. They use a microphone-speaker closed system that measures the frequency response of the microphone and use this measurement to estimate its condition [10]. This technique was tested outdoors as part of the IDEA environmental measurement network and indoors in controlled laboratory conditions.

The following section presents an overview of the sensor's design requirements and details of the casing and software design.

## 2. Design

Our previous work on low-cost environmental sensors determined that commercial off-the-shelf microphone capsules—typically used in consumer electronics—can be used for environmental noise monitoring. These microphones reported small additional averaged errors (no more than 1 dBA) during outdoor tests when compared to Class 1 reference microphones. However, their observed failure rates were significantly higher—some failed in just a couple of months. Consequently, we concluded that quick and reliable sensor failure identification techniques were needed to use low-cost microphones in environmental sensor networks [1]. Building on top of these results, our next task was to develop a low-cost noise measurement sensor capable of quickly and reliably detecting a failure and thus suitable for large-scale deployment in an environmental sensor network. To develop such a sensor we identified two main design requirements:

1. The sensor must be capable of actively testing its condition periodically or on demand. This implies:
  (a) Adding an active component to the sensor—for example a speaker—to generate a test stimulus.
  (b) Triggering the stimulus on demand or on schedule and sample the sensor's response to the stimulus.
  (c) Comparing the sensor's response to the stimulus with a reference *good* response and quantifying this comparison with a value ranging from 0 (no similarity) to 1 (exact match).
  (d) Finding a threshold in the quantification value to classify the sensor's stimulus response into *pass* or *fail*.
  (e) Reporting the result of the test via the sensor network and triggering an alert if necessary.
2. The sensor must be low-cost and capable of outdoors deployment in large numbers. This implies:
  (a) Using low-cost off-the-shelf electronic components to build the sensor.
  (b) Designing a casing for the sensor's components that facilitates environmental monitoring while providing basic protection from the environment.

**Figure 1.**
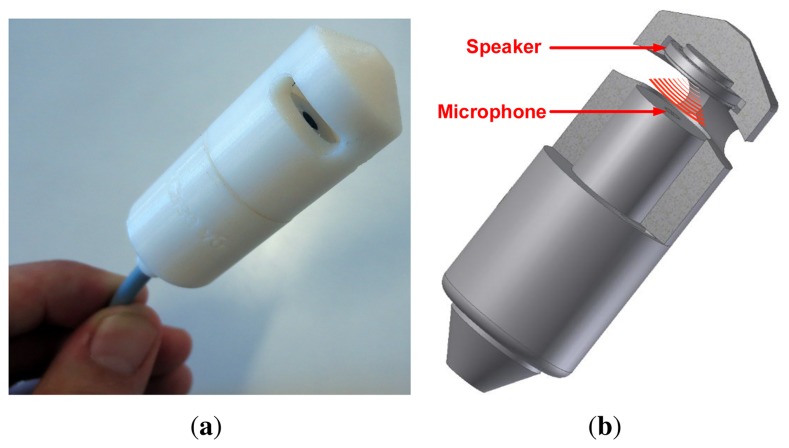
The self-testing noise measurement sensor is a simple combination of a microphone and a speaker embedded in a protective casing. Via audio frequency sweeps, the speaker enables an active test of the microphone's condition and indirectly of the environmental data quality. (**a**) prototype; (**b**) diagram.

Keeping these requirements in mind, the proposed noise measurement sensor consists of a microphone and a speaker facing each other inside a protective casing (Figure 1). The noise sensor then connects to a network node equipped with a sound card with enough computing resources to process audio and relay data over a network connection. The microphone is driven by the node's sound card's input and the speaker is driven by the sound card's output. This simple design uses the same type of microphone capsules already tested for environmental noise monitoring in our previous work and low-cost off-the-shelf speakers typically found in headphones.

We decided not to include signal processing hardware in the sensor design—for example a DSP chip—to maintain a low-cost per unit. Therefore, all online sensor data analysis (specifically requirements 1b-e) are delegated to the sensor node. The following two subsections present the casing design and the accompanying software modules running on the sensor node that drive the active testing functionality.

### 2.1. Casing

The casing design is directly constrained by requirement 2b described in the previous section: the casing must facilitate environmental monitoring while providing protection. More specifically, the casing design must have the following characteristics:

1. *Minimal impact to noise monitoring:* The main purpose of the device is to monitor environmental noise, the addition of the self-test feature must have as little impact as possible to the monitoring task.
2. *Outdoor resistance:* The device will be deployed outdoors and it must be able to withstand wind and rain.
3. *Easy to install and replace:* It is expected that the noise sensor might need to be replaced during the lifetime of the node, so its design must foresee an easy procedure for replacement while in the field.
4. *In the field calibration:* It is expected that this device might need to be calibrated while in the field. The design must support an easy way to calibrate the microphone capsule with a standard $\frac{1}{2}$ inch acoustic calibrator.

**Figure 2.**
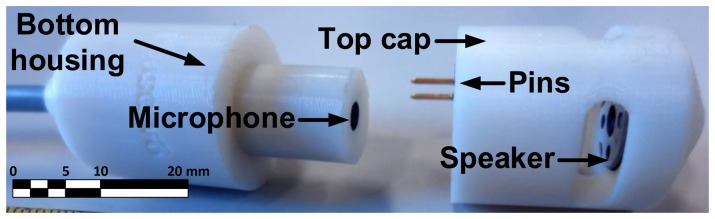
The self-testing noise measurement sensor is divided in two parts: a **bottom housing** that encloses the **microphone** capsule and its circuitry and a **top cap** with an embedded 13 mm **speaker.** The top cap fits on top of the bottom housing placing the speaker just 6 mm from the microphone capsule. The speaker audio signals are connected to the bottom housing and then to the main connector cable via the pair of metallic **pins** seen in the picture.

To implement these characteristics, several prototypes were tested and Figure 2 shows the components of our sixth and last prototype. In this prototype, the *Top Cap* covers the microphone and houses the speaker while the *Bottom Housing* contains the microphone capsule and its circuitry. The bottom housing is constrained by the dimensions of the standard acoustic calibrator. Its top must be cylindrical with a diameter of 13 mm and a height of 15 mm.

**Figure 3.**
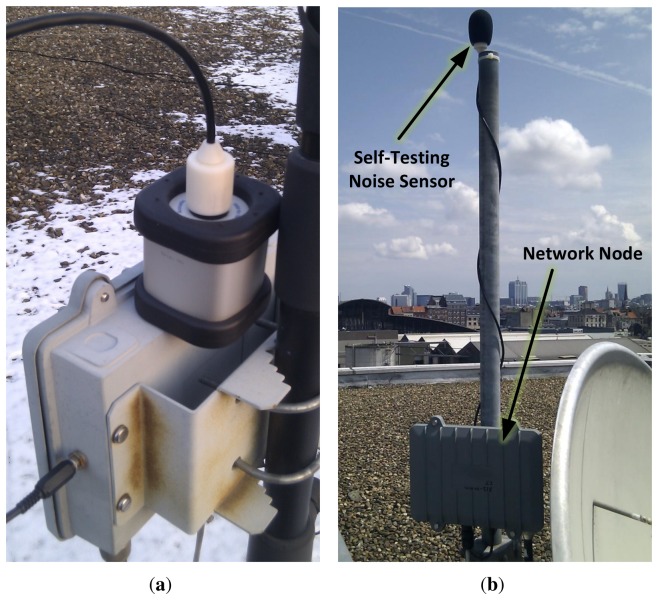
The self-testing noise measurement sensor has been tested outdoors for several months in temperatures ranging from −13 to 38 °C. (a) The casing design has a removable top cap that exposes the $\frac{1}{2}$ inch cylinder with the microphone capsule on top, this allows easy in the field calibration; (**b**) Here, a measuring station (node + sensor) is recording the environmental noise of a busy market in Brussels, Belgium. The sensor prototype is seen here covered with a windscreen to protect it from the weather.

Figure 3a shows the device in the field with the top cap temporally removed to allow for calibration. During normal operation, the sensor is deployed as shown on Figure 3b, with the top cap on (also see Figure 1a) and a windscreen to protect the microphone-speaker system from the weather. It connects to a network node (gray boxes in Figure 3) via a single 4-core cable and a mini-XLR connector. This connector was selected after testing several audio connectors because it allows easy device replacement while providing a good outdoors profile (low noise and mechanical stability). Moreover, mini-XLR is a relatively low-cost component when compared with professional outdoor connectors.

Additionally, to avoid interfering with the sensor's environmental noise measurements, the top cap was designed to avoid air resonance and noise attenuation effects. This was done by enlarging the side holes of the top cap and diminishing the size of the internal cavity between the microphone and the speaker.

### 2.2. Software Modules

All online test data analyses were delegated to run on the sensor node, therefore we developed a group of software modules that implemented the following functionality:

triggering a stimulus, on demand or scheduled, (*requirement 1b*)
sampling the sensor's stimulus response, (*requirement 1b*)
quantifying the response, (*requirement 1c*)
and lastly reporting the result. (*requirement 1e*)

Additionally, due to the remote, difficult to reach nature of a sensor node deployment in an environmental network, the presence of the self-test software must not interfere with the data collecting task of the sensor node and must support seamless installation, update, and removal procedures.

**Figure 4.**
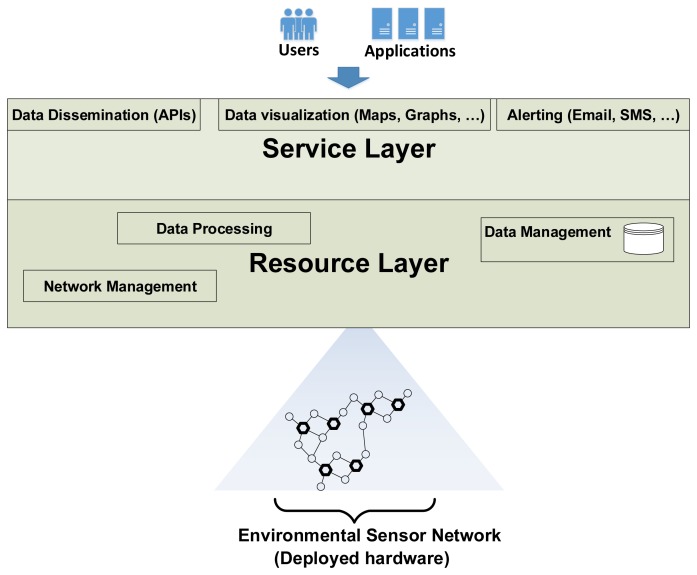
The IDEA Environmental Measurement Cloud, the context where our sensor was tested, is divided in two layers: a *Service Layer* and a *Resource Layer*. The Service Layer is the front-end to the environmental network's users and third party applications while the Resource Layer manages the network's hardware and data resources.

To accomplish this, the self-test software modules must integrate into the currently installed software infrastructure in the IDEA project. This infrastructure, collectively known as the IDEA Environmental Measurement Cloud, is divided in two layers (Figure 4): a *Resource Layer* where the network hardware and data resources are managed and a *Service Layer* where web services for data access and event alerting are hosted (for more details refer to [11]). Except for alert triggering, all software modules implementing the self-test functionality are hosted in the resource layer.

The IDEA environmental network (part of the resource layer in Figure 4) is a sensor network focused on monitoring noise pollution in urban areas. It consists of approximately 40 sensor nodes deployed in Ghent, Brussels and Antwerp (for more details see also [12]). Typically, nodes are installed in building facades or roofs—from where they draw power and Internet access—near interesting noise polluting sources such as streets, highways, train lines and so forth. All sensor nodes are PC Engines ALIX 3d3 Single Board Computers (SBC); they have a 500 MHz AMD processor, 256 MB RAM, 4 GB Compact Flash permanent storage, and an average power consumption of 5 W [13]. They connect to the IDEA environmental network directly via Internet using opportunistic access—depending on the node location, for example public WiFi, building Ethernet, or a GPRS network. These characteristics allow them to run lightweight versions of the Linux operating system and the JAVA platform. Each sensor node uses the OSGi framework to implement core functionality such as data collection, data compression, remote administration and remote monitoring.

**Figure 5.**
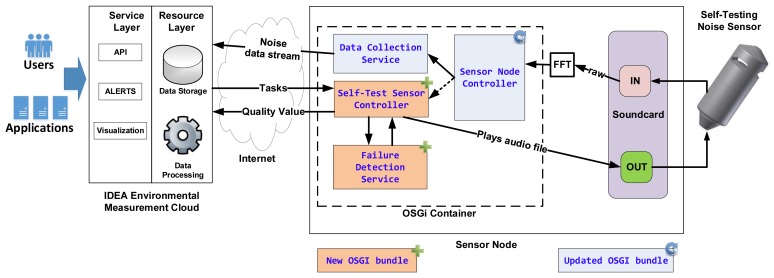
The software modules that drive the self-test functionality run on-board the sensor node. After a self-test, the *Self-Test Sensor Controller* module sends a quality value to the IDEA Environmental Measurement Cloud where the value is stored and decisions such as alerting are taken.

OSGi (Open Services Gateway Initiative) is a technology whose mission is to enable the deployment of services over local and wide area networks and devices [14]. In OSGi, software modules (called bundles) can be installed, updated or removed remotely. Each bundle implements one set of functionality and may use services offered by other bundles to perform its job. Figure 5 shows a simplified diagram of the bundles that implement the self-test functionality: the *Self-Test Sensor Controller* bundle and the *Failure Detection Service* bundle.

**Figure 6.**
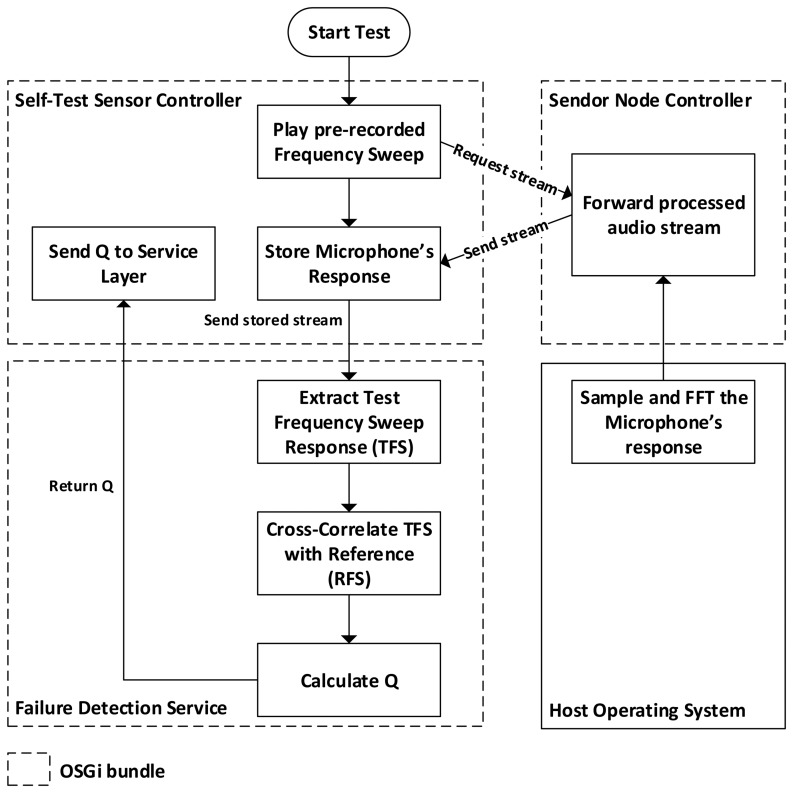
This flow diagram describes the complete process—lasting around 16 s—of a self-test. Test triggering and reporting is performed in the *Self-Test Sensor Controller* bundle while sensor quality quantification is performed in the *Failure Detection Service* bundle.

The *Self-Test Sensor Controller* bundle implements the self-test triggering and scheduling functionality; during a self-test it plays an audio file to the speaker and receives the microphone's feedback. This feedback is then sent to the *Failure Detection Service* bundle where a quality value is calculated and sent back to the *Self-Test Sensor Controller* bundle. This bundle then sends the value over to a remote server where it will be stored and used by the IDEA Service Layer to report the sensor's condition and generate an alert if necessary (Figure 6).

The *Self-Test Sensor Controller* and *Failure Detection Service* bundles encapsulate all the functionality needed to drive the Self-Test noise measurement sensor. The *Self-Test Sensor Controller* implements triggering, scheduling, and sampling (requirement 1b) and reporting (requirement 1e), while the *Failure Detection Service* implements quantification (requirement 1c). These two bundles are not essential to the main task of the sensor node (noise data collection) and can therefore be installed, updated, and removed without disturbing the environmental network. The *Failure Detection Service* bundle was designed as a service interface whose implementation can be easily exchanged to test different failure detection procedures.

Finally, the IDEA Service Layer, via a RESTful API, provides an interface to our self-testing sensor to third party applications. These applications, e.g., a map visualization, could, via an API call trigger a self-test and via another API call, query the sensor's quality value.

## 3. Sensor Quality Quantification

The task of the *Failure Detection Service* bundle running on-board the sensor node is to quantify the sensor's stimulus response with a quality value *Q* ranging from 0 (fail) to 1 (pass). To accomplish this, it must use a failure detection procedure. In this paper, we propose a procedure based on the microphone capsule's response to a frequency sweep generated by the embedded speaker.

Traditional free-field tests that gauge the quality of environmental noise sensor components (microphone capsule, windscreen, casing, *etc.)* use a frequency sweep signal. This signal excites the noise sensor in a given range of audio frequencies and, by measuring the sensor's response, provide an estimation of the system's transfer function and indirectly its condition [10,15,16].

The *Failure Detection Service* bundle introduced in Section 2.2 implements a procedure that by measuring the microphone's response to a pre-recorded frequency sweep returns a quality value ranging from 0 to 1 (Figure 6). This procedure estimates the quality of the sensor by following these steps:

1. *Train:* Obtain an initial reference frequency sweep *RFS* response when the sensor is in a *healthy* state.
2. *Test:* At a later time, obtain a test frequency sweep *TFS* response while the sensor is deployed in the field.
3. *Correlate:* Compare *RFS* and *TFS* by doing a cross-correlation of the two signals. Normalize the result to produce a value ranging from 1 (exact match) to 0 (no correlation).

The following subsections describe the extraction of the frequency sweep response from noise measurements and the use of cross-correlation to generate a sensor quality value.

### 3.1. Frequency Sweep Extraction

Frequency sweeps are normally used as an excitation signal to characterize audio systems in controlled laboratory environments [10,16]. This controlled environment together with high-end test equipment allow for accurate noise-free characterizations of Class 1 and Class 2 Sound Level Meters (SLM) within the IEC 61672-3 periodic tests standard [17]. Our frequency sweeps' tests are performed with low-cost components outside of controlled laboratory conditions (outdoors). While these conditions rule out IEC 61672-3 compliance, to insure a minimum level of test reliability, the effects of uncontrolled environmental noises must be taken into account.

**Figure 7.**
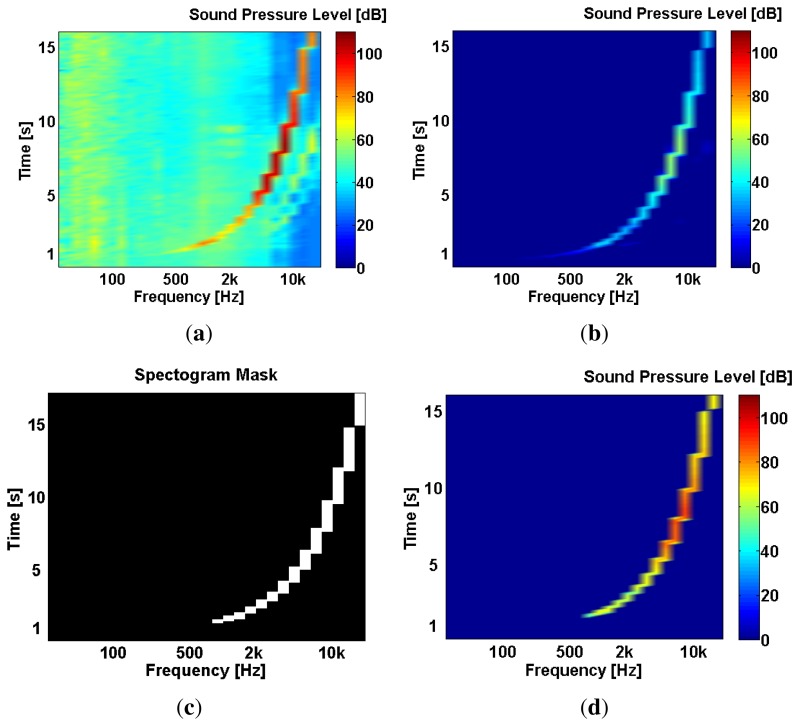
During a self-test, a 16 s frequency sweep is played by the speaker and then captured by the microphone. The microphone's output is first converted to the frequency domain (1/3 octave bands 8 Hz format) and then sent to the Failure Detection bundle. This bundle extracts the frequency sweep from the background environmental noise in four steps which are shown here in four 16 seconds frequency spectrograms. (**a**) Test output (*TO*); (**b**) Filtered output (*TO_f_*); (**c**) Test output Mask *M*; (**d**) Frequency sweep (*FS*).

The self-testing noise sensor produces a 16 s frequency sweep (ranging from 80 Hz to 20 KHz) that is captured by its microphone and used as an estimation of the sensor's frequency response. To effectively use this frequency sweep while in the field, the environmental background noise must be removed from the microphone's response. Given that the speaker is placed just 6 mm away from the microphone capsule, the frequency sweep excitation signal can be extracted from the microphone output by assuming this: *the power contribution of the frequency sweep will be higher than the power contribution of the environmental noise*. Using this assumption, the frequency sweep signal can be extracted from the 16 seconds microphone's response using the following steps:

1. Capture the microphone's 1/3 octaves output during the self-test, let's call it **test output** *TO* (Figure 7a). Use the first second of *TO* to estimate the current environmental noise level *EN*. During this first second, the actual sweep has not started yet.
2. Substract *EN* (and a small but tunable *δ* value) from *TO* to obtain a **filtered test output** *TO_f_*. The frequency sweep in *TO_f_* is strongly attenuated, but isolated from background noise (Figure 7b).
  $$TO_{f}=TO-\left(EN+\delta \right)$$
3. Using *TO_f_*, a mask *M* (Figure 7c) is created to extract the original frequency sweep:
  $$\begin{array}{cc} M= & \{\begin{array}{cc} 1, & \text{if}\;TO_{f}>0\\ 0, & \text{if}\;TO_{f}\leq 0 \end{array} \end{array}$$
4. Finally, the original **frequency sweep** response *FS* (Figure 7d) is extracted from its background noise by multiplying *TO* and *M*:
  FS=TO⋅M

### 3.2. Cross-Correlation Quality Value

The basic assumption behind a frequency sweep test is that any deviation from the original frequency response of the noise sensor indicates a possible system degradation or failure. The Reference Frequency Sweep (*RFS*) represents the original healthy-state frequency response and a Test Frequency Sweep (*TFS*) represents the frequency response during a test. The similarity between these two functions can be determined by using a cross-correlation.

Cross-correlation is a measure of similarity between two signals. The definition of the cross-correlation between two discrete signals *f* and *g* in a finite interval *N* is presented in Equation (1).

(1) $$\left(f⋆g\right)\left[m\right]=\{\begin{array}{ll} \sum_{n=0}^{N-m-1}f*[n]\;g[n+m], & \text{if}\;m\geq 0\\ \left(f⋆g\right)[-m], & \text{if}\;m<0 \end{array}$$

Equation (1) tends to produce rather large values, also known as correlation coefficients. These coefficients can be scaled down, and are therefore handled with smaller data types, by normalizing the cross-correlation as shown in Equation (2).

(2) $${\left(f⋆g\right)}_{\text{unbiased}}[m]=\frac{1}{N-\left|m\right|}\left(f⋆g\right)[m]$$

(3a) $$Rc={\left(\text{RFS}⋆\text{TFS}\right)}_{\text{unbiased}}[m]$$

(3b) $$Ra={\left(\text{RFS}⋆\text{RFS}\right)}_{\text{unbiased}}[m]$$

The coefficients of the cross-correlation between *RFS* and *TFS* (Equation (3a)) quantify the level of similarity between the two signals and the auto-correlation coefficients (Equation (3b)) provide us a scale for this similarity The lag or position of the maximum value coefficient represents the lag or phase where the two signals match.

**Figure 8.**
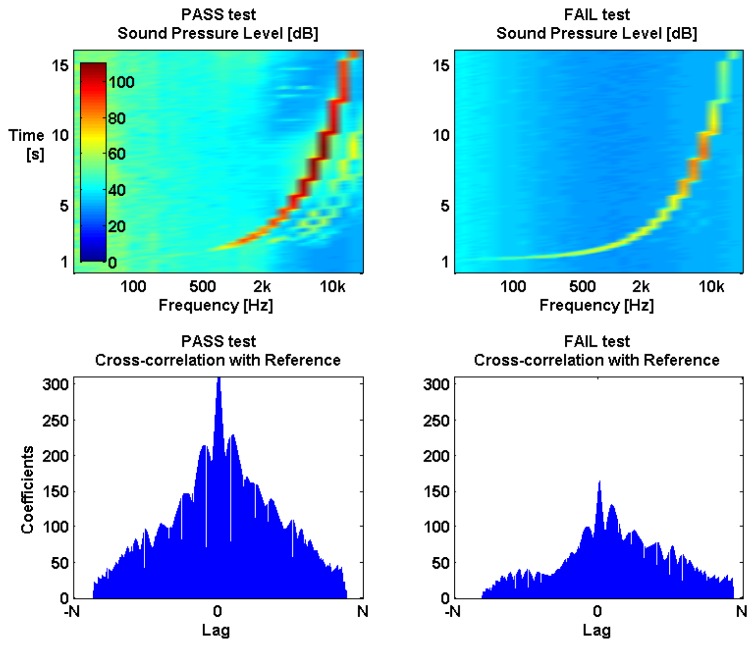
Two frequency sweep tests are shown here: one during a healthy sensor state−PASS test−and the other during a sensor failure−FAIL test. During a PASS test, the cross-correlation between the test frequency sweep (*TFS*) and the sensor's reference frequency sweep (*RFS*) produces large coefficients with time lags close to 0. By comparison, during a FAIL test, cross-correlation coefficients will be smaller and possibly time shifted. Notice the conspicuous absence of background environmental noise in the FAIL test spectrogram, providing an extra clue that something is amiss with the noise sensor.

During a healthy sensor state, *RFS* and *TFS* should be identical and therefore the cross-correlation coefficients *Rc* should be identical to the auto-correlation coefficients *Ra* with a lag near 0 (PASS test in Figure 8). Based on this, Equation (4) provides then a quantification *Q* of the deviation between *Rc* and *Ra* with a value ranging from 0 to 1, where *N* is the sample size and *L_max_*_(_*_Rc_*_)_ is the lag of the maximum value of Rc. Equation (4) will approach 1 as *RFS* and *TFS* match and will approach 0 as RFS and TFS diverge either in phase or in amplitude (FAIL test in Figure 8).

(4) $$Q=(1-\frac{\left|\max(Ra)-\max(Rc)\right|}{\max(Ra)})\cdot (1-\frac{\left|L_{\max}(R_{c})\right|}{2N-1})$$

Equations (3a), (3b) and (4) are implemented by the *Failure Detection Service* bundle on-board the sensor node to produce a value of *Q* after each test. This value is then transmitted by the *Self-Test Sensor Controller* to the IDEA Service Layer and used as an estimation of the quality of the noise measurement sensor.

## 4. Sensor Quality Classification

Sensor quality classification—determining if a sensor is failing or not—is not performed on-board the sensor node because classification is only needed to generate sensor failure alerts. Alerts are generated in the IDEA Service Layer and therefore it is there where the reported *Q* values are classified.

The value of *Q* presented in the previous section is an estimation of the condition of the microphone-speaker system. It quantifies the sensor quality but it does not yet classify it as either fail or pass−with the exception of the extreme values of 0 or 1. To classify a *Q* value as pass or fail, it is necessary first to establish a threshold value for *Q*. This value was determined experimentally in a controlled lab environment and tested in a real outdoors deployment in the IDEA sensor network. This section presents and discusses our experimental results and evaluates the effectiveness of *Q* and a candidate threshold value *T*(*n*) to detect sensor failures.

### 4.1. Experimental Setup

To evaluate the accuracy of *Q* as a sensor failure estimator, we performed a set of tests in a controlled laboratory setting indoors. Additionally, we equipped six IDEA sensor nodes with the self-testing noise sensor. Four of these nodes were deployed outdoors, functioning as an environmental noise monitoring station and as a test-bed for our self-testing noise sensor. They were exposed to the usual weather conditions (rain, snow, hail, strong winds) found in northern Europe with temperatures ranging from − 13 to 38 °C (Figure 3) for a duration ranging from 3 months to 6 months.

All self-test noise sensors were assembled with off-the-shelf speakers and microphone capsules (see Table 1). All casings were 3D printed using Acrylonitrile Butadiene Styrene (ABS) plastic.

The experiments were divided in two parts: laboratory tests and outdoor tests. The laboratory tests were performed indoors and were mainly designed to measure how *Q* changes in the presence of known controlled failure conditions. The outdoor tests were performed in the field to determine how *Q* changes when exposed to real failure conditions caused by the wear and tear of an outdoors environment and evaluate the effectiveness of a candidate threshold value to classify sensor failures using an independent sensor failure classification technique.

### 4.2. Laboratory Tests

For the laboratory experiments, we exposed several self-testing noise measurement sensor prototypes to the following failure conditions:

1. *damaged microphone capsule*, the microphone component was punctured and heated,
2. *damaged speaker*, the speaker component was disconnected,
3. *obstructed microphone capsule*, an obstruction was placed on top of the microphone capsule and
4. *wet microphone capsule*, the microphone capsule was submerged in water prior to a self-test.

All these failure conditions, except for the damaged speaker, are known to degrade the noise data collected by the microphone capsule.

We built 20 self-test noise measurement prototypes to perform these experiments, 10 were built with the MC component and 10 with the MK component (see Table 1). Before exposing these devices to the enumerated failure conditions, their *Q* values in a healthy state was recorded and averaged.

**Figure 9.**
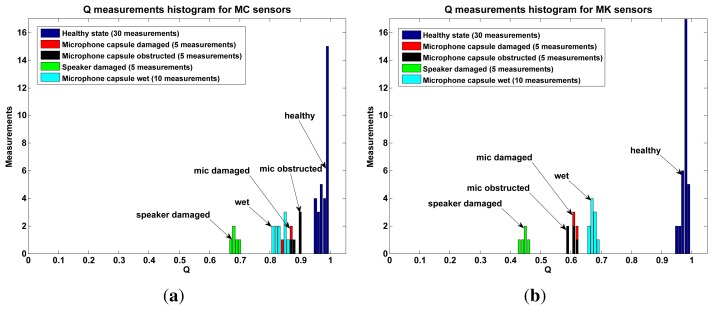
We subjected several MC and MK self-test noise sensors to different failure conditions: wet, broken and obstructed microphone capsules and broken speakers. The produced Q values for the MK sensors were more sensitive to these failure conditions in comparison with the MC sensors. (**a**) MC self-test sensors *Q* values in failure conditions can get rather close to the *healthy* median (*M_h_* = 0.9844, blue bars); (**b**) MK self-test sensors *Q* values in failure conditions are farther away from the *healthy* median (*M_h_* = 0.9789, blue bars).

Figure 9 shows a histogram of the *Q* values obtained for healthy devices (blue bars) and for damaged devices. While these results show that *Q* is indeed sensitive to failure conditions, the sensitivity is remarkably different between MC and MK sensors. This difference is also reflected in Table 2 where the minimum distance between *Q_f_* (failed state values) and *M_h_* (median healthy state values) is 32 times the standard deviation of *Q_h_* (healthy state values) for MK while only 5 times for the MC sensors.

The indoor experiments show that in principle it is possible to use the value of *Q* to estimate a sensor failure. By setting a threshold of 5 standard deviations below the median of *Q_h_*, a possible failure can be estimated for MC and MK sensors. This threshold is safe for MK based noise sensors, however it might produce false positives in MC sensors.

### 4.3. Outdoor Tests

The laboratory tests showed that the value of *Q* can be used as a metric to estimate the quality of a noise sensor. However, environmental noise sensors are mostly placed outdoors and not in controlled laboratory environments. To estimate the practical usefulness of *Q*, we placed four sensor nodes outdoors—each node equipped with one self-testing noise measurement sensor—and tasked them to measure *Q* hourly. Table 3 lists all deployed nodes plus a small control group placed indoors. Test Node 1 was placed on top of a roof near an outdoor market while Test Nodes 2–4 were placed in the same location—on top of a roof facing a street and a canal, about 150 m far from Test Node 1.

Test Nodes 5 and 6 (control nodes) produced *Q* values tightly packed around their median of 0.98 with a standard deviation of around 0.001 for both nodes. Because they were not exposed to harmful environmental conditions, they reported no failures and their measured *Q* values stayed close to 1 during their entire operational period.

**Figure 10.**
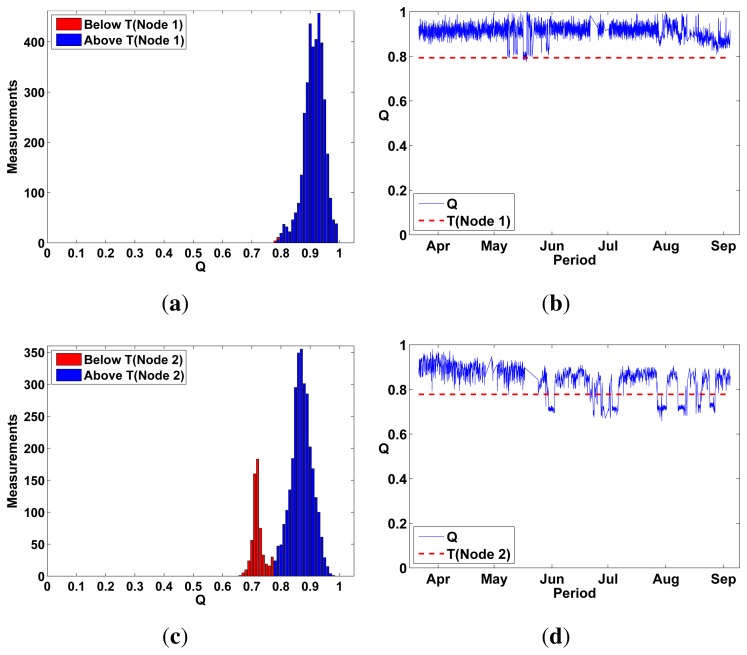
Test Nodes 1 and 2 were placed outdoors for a period of 6 months and measured *Q* hourly. *Q* values for Test Node 1 didn't vary enough to detect any sensor failure. However, *Q* values for Test Node 2 had a clearly bimodal distribution where *fail* values could be clearly discriminated from *healthy* values by using *T*(*n*). (**a**) Q distribution Test Node 1 (MC); (**b**) Q values Test Node 1 (MC) 6 months; (**c**) Q distribution Test Node 2 (MC); (**d**) Q values Test Node 2 (MC) 6 months.

**Figure 11.**
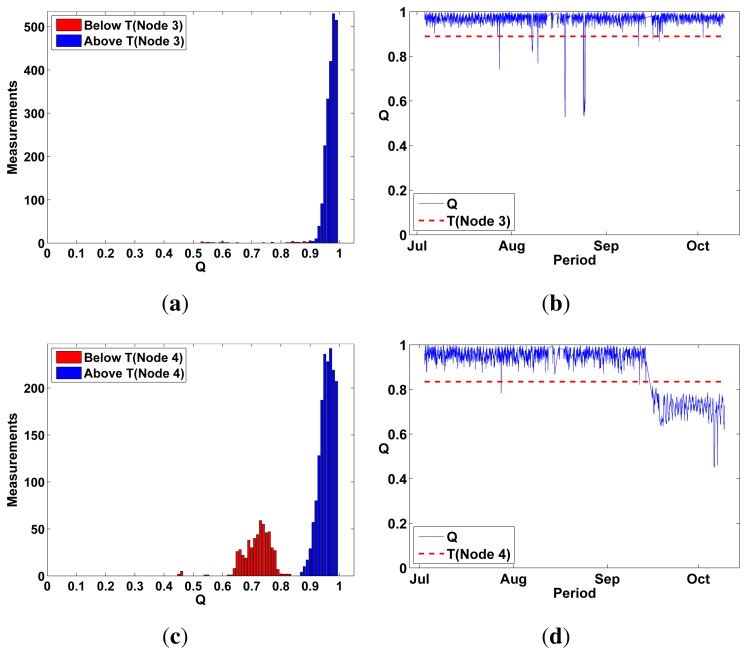
Test Nodes 3 and 4 were placed outdoors for a period of 3 months and measured *Q* hourly. *Q* values in Test Node 3 have transient *fail* events (b) where they fall well below *T*(*n*). Test Node 4 drifts below *T*(*n*) (d) at the end of its operating period suggesting a permanent sensor failure, (**a**) Q distribution Test Node 3 (MK); (**b**) Q values Test Node 3 (MK) 3 months; (**c**) Q distribution Test Node 4 (MK); (**d**) Q values Test Node 4 (MK) 3 months.

On the other hand, *Q* values for Test Nodes 1–4 had a much wider *Q* distribution, even multimodal, suggesting the detection of failure events (Figure 10a,c and Figure 11a,c). To classify *Q* values as either *pass* or *fail*, we defined a threshold *T*(*n*) individually for each self-testing sensor (represented by *n*) by using the median and standard deviation of their first 150 *Q* values. These initial values can be safely assumed to be measured during a healthy sensor state. *T*(*n*) is defined as:

$$T(n)=M_{150}(n)-5\sigma_{150}(n)$$

where M_150_(*n*) is the median of the first 150 *Q* values measured outdoors for a sensor *n* and σ_150_ is the standard deviation of these values. *T*(*n*) classified only outlier values as *fail* in MK type sensors (Figure 11).

MC type sensors produced no clear outlier values (Figure 10) and *T*(*n*) was therefore unable to clearly classify Q values as *pass* or *fail*. This was partly due to the lower quality of MC sensors; we observed that they were sensitive to crosstalk during self-tests and therefore produced artificially high frequency sweep responses during failure conditions.

#### 4.3.1. Independent Sensor Failure Classification

The threshold *T*(*n*) seems like a good candidate to classify *Q* values as *either pass* or *fail*. However, its effectiveness to detect sensor failures can only be evaluated by independently determining when noise sensors actually fail.

One method to estimate sensor malfunctions is to use our domain knowledge of environmental noise to detect patterns and anomalies in the sensor data output [2,18]. A well-known pattern in urban environmental noise is the *diurnal pattern* [19], the cyclic up and down of the average noise levels caused by the cycle of human activity in a city (work and commute during day, sleep at night). Healthy noise sensors deployed in an urban environment are expected, on average, to produce this pattern in their data output. Any long-term interruption of this pattern can be used to estimate an environmental noise sensor malfunction.

**Figure 12.**
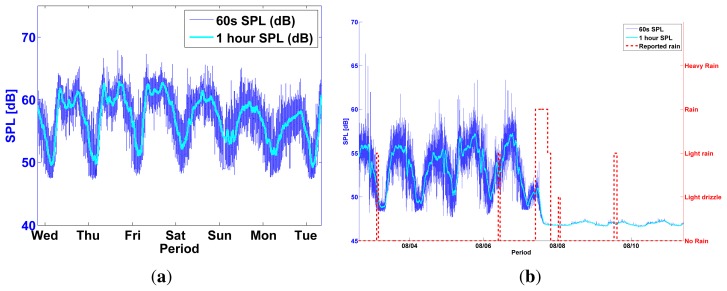
All outdoor test nodes produced a typical urban noise diurnal pattern [19] during healthy conditions (**a**). A sensor failure becomes evident with a visible interruption of the diurnal pattern in the sensor's output (**b**). Superimposed local weather data suggest that overexposure to rain might be the cause of most sensor malfunctions. (**a**) Diurnal pattern Test Node 2 (MC); (**b**) Sensor failure Test Node 2 and weather (MC).

Figure 12a shows the diurnal pattern produced by Test Node 2 during healthy sensor conditions. This diurnal pattern was visibly interrupted several times during the test period of all outdoor sensors and, with the help of local weather data, we could determine that an overexposure to rain caused most sensor malfunctions (Figure 12b). Weather data—rain levels—were measured from a weather station in Zaventem, Belgium. They were provided by WeatherSpark.com.

**Figure 13.**
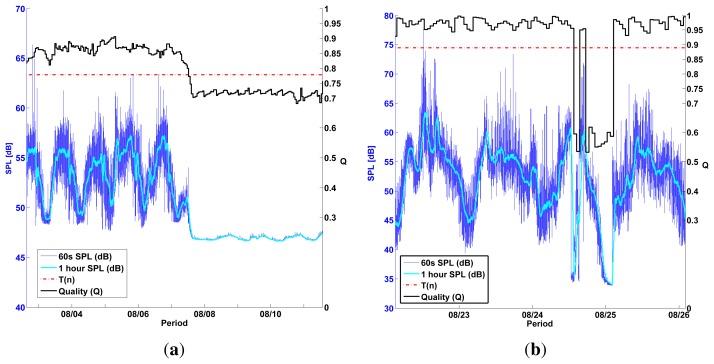
Two failure scenarios are shown here superimposed with the measured quality (Q). *Q* drops below the threshold *T*(*n*) immediately after a sensor failure, proving its capacity to detect a failure condition. (**a**) Sensor failure Test Node 2 and Q (MC); (b) Sensor failure Test Node 3 and Q (MK).

Figure 13 shows two examples of how *Q* drops below the threshold *T*(*n*) during a sensor failure scenario. Both failure scenarios are an example of the *CONSTANT* malfunction, as defined in [18], where a sensor output is inexplicably constant with a variance close to 0. These events are correctly classified as *fail* by the measured *Q* value.

**Figure 14.**
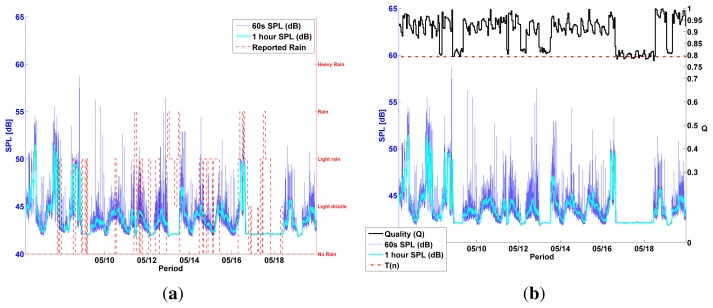
Seven failure scenarios in Test node 1 are shown here for a period of two weeks. Plot (**a**) shows the sensor's output superimposed with weather data, suggesting that rain caused all observed failures. A plot of the same period, superimposed with *Q* values (**b**), shows that *Q* barely reaches *T*(*n*) during the observed sensor failures. (**a**) Sensor failures Test Node 1 and weather (MC); (**b**) Sensor failures Test node 1 and Q (MC).

Figure 14 shows a few examples of observed sensor failures in Test node 1. *Q* values measured in this node drop during sensor failures but barely reach *T*(*n*). Here, *Q_f_* values appear to be too close to the healthy median *M_h_*, probably due to crosstalk caused by faulty wiring or by the microphone capsule.

**Figure 15.**
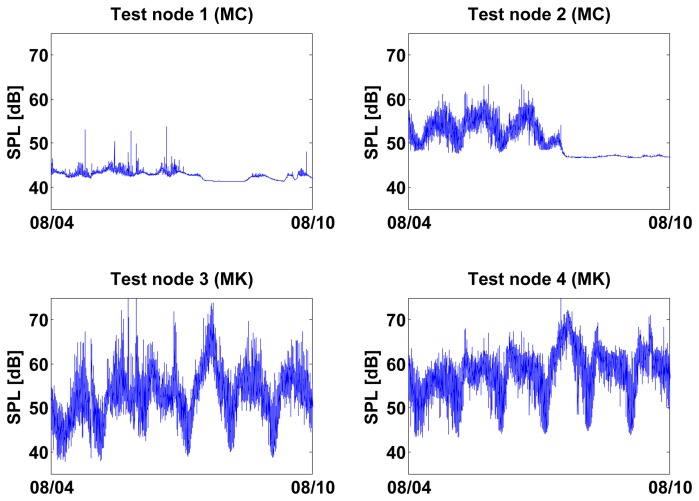
The same one week period depicted in Figure 12b is shown here but for all outdoors nodes. Between Test Nodes 2–4, only Test Node 2 breaks its diurnal pattern, despite all three being deployed at exactly the same location. Test node 1 was deployed in a different location where the average noise levels were lower.

Additionally, spatial correlation could be used to independently confirm whether a sensor has failed [18], specially in high-spatial density sensor networks [2]. This is illustrated in Figure 15 where the same time period is represented for all outdoor nodes. For testing purposes, Test Nodes 2−4 were placed at the same outdoor location, however only Test Node 2 breaks the diurnal pattern with an apparent CONSTANT fail condition. This inconsistency suggests that the flat constant behavior in Test Node 2 is a malfunction and not an unlikely “quiet” period of noise pollution.

Table 4 shows a summary of how effective was *Q* and the threshold *T*(*n*) to classify all observed sensor malfunctions as *fail* in our outdoor deployments. Except for malfunctions in Test Node 1, all other malfunctions were correctly classified as *fail*. A few *fail* classifications were false positives or false alerts, possibly due to unusually loud environmental noises interfering with the self-test. However, most false alerts are transient, produced by only one *Q* measurement, and could be easily weeded out by scheduling a second self-test.

## 5. Conclusions

All sensors equipped with the middle-range value Knowles microphone capsule (MK type) were able to successfully detect and report all of their failures while deployed outdoors for several months. Their quality *(Q)* values showed a clearly multimodal distribution in laboratory and outdoor tests alike, suggesting that *fail Q* values can be clearly distinguished from *pass* values. While they generated a few false positives during their outdoor tests, most of these false alerts were transient and could be eliminated by triggering a new frequency sweep.

Compared with the total cost of ownership of a large-scale environmental network, the extra cost of the self-testing functionality is negligible. A self-testing noise measurement sensor, equipped with an MK type microphone capsule, costs €50 or less, depending on the production volume. This sensor is already capable of effectively monitoring environmental noise while providing early warnings for its replacement or for the integrity of the data it produces. Additionally, if the environmental network IT infrastructure is built on top of a modular framework, as is the case of the IDEA network, the cost of updating an already deployed sensor node firmware to support our failure detection algorithm is also negligible and with no impact to the network environmental monitoring task.

### 5.1. Failure Characterization

An early goal of the self-testing noise measurement sensor design was to not only detect a failure but also to characterize it. Failure characterization is the classification or identification of the nature of the system malfunction. It answers questions such as: is the microphone capsule wet? Or frozen? Or is the speaker damaged?

**Figure 16.**
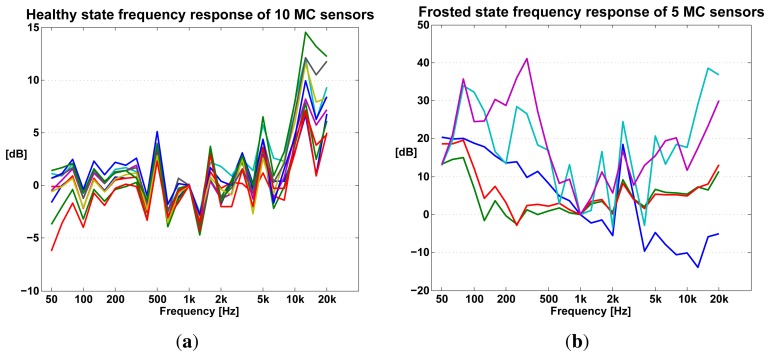
Plots a and b show the frequency response of MC sensors when new (**a**) and when exposed to frosting (**b**) in a weather chamber. The measured frequency response of healthy MC sensors, while not ideally flat, is reproducible. However, the same capsules, when frosted, output a non-reproducible frequency response, precluding the use of the frequency response to characterize a failure condition. The frequency response plots presented here were obtained using a high-end acoustic measurement chain. (**a**) Frequency response of healthy microphone capsules; (**b**) Frequency response of frosted microphone capsules.

Our hope was to find patterns in the sensor's frequency response during induced malfunctions in a weather chamber, as was done in [16]. However, we had to abandon this goal as the frequency response varied almost randomly during failure conditions, for MC (Figure 16) and MK sensors alike.

Nevertheless, analyzing other more complex temporal or spectral features of the sensor's data output during an active test may provide a more accurate error estimation and possibly failure classification. These techniques typically demand computational resources not available in sensor networks, specially when online processing is required. However, advances in the miniaturization of electronic devices is quickly changing this. For example, in [20], the authors classified sound samples in a mobile phone by extracting features such as Zero Crossing Rate (ZCR) and Spectral Flux (SF) plus a few others. They successfully proved that online sound classification in resource constrained devices is possible.

Our design can only detect a failure condition in the microphone-speaker system, therefore we envision this capability as a complement to passive error classification techniques. Once our sensor raises a malfunction alert, intelligent sensor malfunction detection techniques [2,18] can be activated to further classify the malfunction alert or discard it as a false positive.

### 5.2. Future Work

Sensors equipped with low-cost Kingstate microphone capsules (MC type), each costing less than €10 to build, presented a higher number of failures and where also less effective in detecting them. Due to the lower quality of the microphone capsule, they were more susceptible to cross-talk interference during a frequency sweep self-test. As a consequence, the self-test had a tendency to measure the cross-talk response instead of the microphone capsule response. Eliminating this effect in future versions (better shielding) could then allow the use of low-range value microphone capsules in our self-testing noise sensor.

Finally, the casing design could still be improved in two aspects: polar response and rain protection. Future prototypes will take into account polar response tests to optimize the top cap design and avoid altering the hlomnidirectionality of the microphone capsules. Additionally, covering the top cap side holes with an acoustically transparent fabric (nylon, latex) could improve the sensor's resistance to rain and wind.

## Figures and Tables

**Table 1.** The components listed here were used to assemble the self-testing noise sensors prototypes. The Kingstate microphone capsule was tested as an environmental noise sensor in [1] and the Knowles microphone capsule has been used extensively for the same purpose in the IDEA network. The last column is the abbreviation used for each component in Sections 4 and 5.

**Component Description**	**Model**	**Avg. Price**	**Abbrv.**
Speaker, ø13 mm, typically used in headphones	Kingstate KDMG13008C-03	€1	S
Electret microphone capsule, ø4 mm, omnidirectional	Kingstate KEEG1542PBL-A	€1	MC
Electret microphone capsule, ø2 mm, omnidirectional	Knowles FG-23329-P07	€30	MK

**Table 2.** The *Q* values presented in Figure 9 can be divided in two categories: healthy state (*Qh*) and failure state (*Qf*). MK sensors have a smaller *σ_h_* and a bigger distance between *Q_h_* values and *Qf* values, making *Q* a more effective estimator for microphone failure in MK sensors.

**Parameter**	**MC**	**MK**
Median *Q_h_* (*M_h_*)	0.9844	0.9789
Standard deviation *Q_h_* (*σ_h_*)	0.0155	0.0091
Maximum observed *Q_f_*	0.9048	0.6860
Distance from maximum *Q_f_* to *M_h_*	5*σ_h_*	32*σ_h_*

**Table 3.** To test the capacity of *Q* to estimate sensor quality in an outdoors environment, four nodes were placed outdoors to measure the environmental noise produced by an urban *feature of interest* for a period of several months. They were equipped with our self-test noise sensors (types MC and MK) which measured *Q* hourly Additionally, two nodes were kept indoors to serve as a control group.

**Description**	**Feature of Interest**	**Placement**	**Sensor Type**	**Duration**	**Q Period**
Test Node 1	outdoor market	outdoors	MC	6 months	1 per hour
Test Node 2	street & canal	outdoors	MC	6 months	1 per hour
Test Node 3	street & canal	outdoors	MK	3 months	1 per hour
Test Node 4	street & canal	outdoors	MK	3 months	1 per hour
Control Node 5	auditorium	indoors	MC	6 months	1 per day
Control Node 6	lab	indoors	MK	3 weeks	1 per hour

**Table 4.** The effectiveness of *Q* is defined as the percentage of actual sensor malfunctions that were correctly classified as *fail* by *Q*. A wrong *fail* classification is defined as a false positive (False +) and a spurious fail classification (only one *fail* measurement) is defined as a transient false positive. Except for test Node 1, all sensor malfunctions were correctly classified by our detection method.

**Node**	**Malfunctions**	**Effectiveness Q**	**False +**	**Transient False +**	**False −**
Test Node 1	16	6.25%	0	0	15
Test Node 2	8	100%	1	3	0
Test Node 3	3	100%	3	5	0
Test Node 4	1	100%	1	2	0
